# Role of Bile Acids and Gut Microbiota in Parenteral Nutrition Associated Injury

**DOI:** 10.36959/487/286

**Published:** 2020-03-02

**Authors:** Chandra Shekhara Manithody, Johan Van Nispen, Vidul Murali, Sonali Jain, Ashish Samaddar, Austin Armstrong, Ajay Jain

**Affiliations:** Department of Pediatrics, SSM Cardinal Glennon Hospital, Saint Louis University School of Medicine, USA

**Keywords:** TPN: Total parenteral nutrition, Bile acid, DC: Duodenal catheters, CDCA: Chenodeoxycholic acid, FGF19: Fibroblast growth factor 19, FXR: Farnesoid x receptor

## Abstract

Total Parenteral Nutrition (TPN) is a life-saving therapy where all nutritional requirements are provided intravenously. While this therapy is essential for individuals unable to process their nutritional needs enterically, significant complications arise such as intestinal failure associated liver injury (IFALD). IFALD includes hepatic steatosis, cholestasis, inflammation, ultimately progressing to cirrhosis and portal hypertension and some patients may need liver transplantation. The exact mechanism underlying this condition is not well understood, but studies have recently suggested that changes in gut microbiota and intraluminal bile acid signaling are known to play a role in the development of IFALD. In enterohepatic circulation with normal enteral nutrition, gut Farnesoid X Receptor (FXR) is activated by bile acids, which triggers the release of Fibroblast Growth Factor 19 (FGF19) into portal circulation. FGF19 serves to regulate intrahepatic bile acid synthesis with enteric nutrition. This signaling pathway is impaired in TPN as studies indicate decreased serum levels of FGF19 in subjects receiving TPN. Finally, gut microbiota is severely altered in TPN due to intestinal hypomobility. The shift in gut microbiota affects our immune response and promotes endotoxins that negatively affect liver function. Targeting the pathways affecting gut microbiota and bile acid signaling has promise in treating TPN associated injuries.

## Introduction

Parenteral Nutrition (PN) refers to the delivery of essential macro and micronutrients intravenously [1,2]. When PN meets all nutritional needs, the process is called Total Parenteral Nutrition (TPN). Since its first use in the 1960s, TPN has provided life-saving nutritional therapy for individuals who are not able to absorb nutrition for various anatomic or functional disorders, and, thus, remains a promising modality for providing nutrition in neonate, pediatric, and adult patients with lost or impaired gut activity [3].

Although the benefits of this therapy remain undisputed, enthusiasm for its use is tempered due to its association with adverse effects. One such complication that may arise is intestinal failure-associated liver injury (IFALD). Although classically acknowledged as a progressive cholestatic liver injury, IFALD can present with signs and symptoms of liver failure, with histologic evidence of steatosis, cirrhosis and portal hypertension, ultimately resulting in liver transplantation in some individuals. Patients with IFALD also experience a disrupted glucose and lipid metabolism [4]. Another common complication with TPN is gut atrophy, often characterized by decreased villi height, crypt depth, and enterocyte migration rates [5,6].

While multiple mechanisms have been proposed, the exact etiology and pathophysiology of TPN associated injury remains elusive. Nevertheless, emerging data suggests that alterations in the gut microbiota, as well as intraluminal bile acid driven signaling, may play a critical role in both hepatic and intestinal injury. Evidence from recent studies also shows that factors occurring during TPN such as compromised intestinal permeability and inflammation, bacterial translocation, and lack of enteral nutrition are all associated with intestinal microbial dysbiosis [7], further strengthening the idea that gut microbes can influence TPN associated pathology.

## Role of Enteral Nutrition

Clinical studies have shown that even small amounts of enteral nutrition can mitigate the negative effects of TPN. In a clinical study evaluating hyperbilirubinemia, a surrogate for hepatic injury with TPN, findings noted that there is reversal of cholestasis only when a majority of nutrition was given through the enteral route [8]. Several other studies have also confirmed the protective role of enteral nutrition [9].

Recent data point to complications in enterohepatic circulation (EHC) of bile acids secondary to an interruption in enteral feeding as a potential driver for TPN associated liver injury [2], underscoring the importance of luminal nutrients and luminal derived signaling in mediating TPN associated injury. Further, a lack of enteral feeding during TPN is hypothesized to disrupt hepato-protective gut originating signals via impairment of bile acid EHC. The novel idea highlighted here is that rather than the specific content of TPN solution, it is lack of luminal content as in TPN therapy which causes alterations in gut-originating signaling and contributes to liver and gut injury [2].

## Enterohepatic Circulation

Normal EHC is dependent upon a rigorously regulated pattern of bile acid synthesis, conjugation, secretion and recirculation, all of which is driven by enteral nutrition. A lack of enteral nutrition during TPN impairs this homeostatic equilibrium. This calls to attention a critical question: If the gut and the liver signal, could disruption of these signaling events drive TPN associated injury?

This question has become particularly important with the discovery of the nuclear receptor Farnesoid X Receptor (FXR) and the characterization of the metabolically active protein Fibroblast Growth Factor 19 (FGF19) [10,11]. Bile acids, specifically chenodeoxycholic acid (CDCA), are known ligands for FXR, which is present in the gut and, maximally, in the terminal portion of the ileum [12,13]. Given the localization of FXR expression, its affinity for bile acids, and the evidence that bile acids regulate gut growth [2], it is plausible that FXR activation, or the lack thereof, may play a key role in TPN associated injury.

## FXR-FGF19 Axis and its Regulation by Bile Acids

Fibroblast Growth Factor 19: During normal enterohepatic circulation with enteral nutrition, bile acids activate gut FXR. In fact, data from both animal studies and cell culture experiments have shown that there is robust activation of FXR in intestinal cells with bile acid introduction [13]. Bile acid activation of FXR in the gut results in the release of the protein Fibroblast Growth Factor 19 (FGF19), which reaches the liver via the portal system to exert metabolic effects [14].

Bile acids and Hepatic Homeostasis: When regular nutrition is being provided, FGF19 regulates intrahepatic bile acid synthesis, which is impaired with TPN therapy via FGF19 signaling suppression of intrahepatic cholesterol 7 alpha-hydroxylase (CYP7A1), a bile acid synthesis rate-limiting step [15].

Now, enterocytic release of FGF19 via gut FXR activation is widely believed to serve as a luminal signal to the liver to regulate intrahepatic bile acid synthesis. Such signaling is impaired with TPN therapy. In fact, with bile duct ligation, a quick and sustained reduction in serum FGF19 levels occurs, which further supports this mechanistic pathway [16].

Importantly, while gut FXR activation results in FGF19 release, this pathway is not active in the liver. This was confirmed by testing tissue-specific roles of FXR. Treatment by FXR agonists resulted in significant repression of CYP7A1 in liver FXR knockout mice, but not in gut FXR knockouts, attesting to the regulation of CYP7A1 by intestinal FXR activation, thus underscoring the importance of luminal signaling [17]. Indeed, animal studies have confirmed that serum FGF19 levels are significantly lower during TPN therapy than the robust levels observed upon enteral nutrition [2].

Additionally, to objectively quantify and assess whether levels of FGF19 were altered with enteral food deprivation and bile acids, serum FGF19 levels were evaluated in human patients treated with the gut FXR agonist, CDCA, or the bile acid sequestering agent, cholestyramine. Hepatic bile acid production was estimated by measuring the serum surrogate 7alpha-hydroxy-4-cholesterin-3-one (C4), which is a known marker for the enzymatic activity of CYP7A1. While cholestyramine resulted in an almost18-fold increase in the serum concentration of C4 with an accompanying 87% reduction in FGF19 levels, bile acid treatment combined with CDCA drove a 250% increase in FGF19 levels and a decrease in serum C4 by 26% [18]. Further corroborating these responses, animal studies have reported significant hepatic injury and gut atrophy upon TPN infusion with improvement upon delivery of enteral bile acid analogs [2,19].

## Lipid and Glucose Homeostasis

The fact that TPN therapy results in glucose and lipid abnormalities is well established. Recent evidence suggests that such alteration might be secondary to altered gut-derived signaling driven by a lack of bile acid mediated gut FXR activation in conjunction with TPN infusion. In fact, recent data provides compelling evidence that FGF19 additionally regulates glycemic and lipid metabolism. In support of this theory, urine studies have shown a significant improvement in diabetes and a reduction in triglycerides in animals receiving an infusion of recombinant FGF19. FGF19 infusion also resulted in an acute decrease in messaging for the leptin receptor and acetyl coenzyme A carboxylase, both of which are key regulators of lipid homeostasis. Large animal studies have also shown that TPN induced insulin resistance and hypertriglyceridemia can be significantly improved with enteral bile acid treatment [2].

## Gut Microbiota and TPN

There has been significant recent interest in critically evaluating the role of gut microbiota as a driver for TPN associated injury. Due to a lack of luminal nutrition, significant alterations in the gut microbiota, as well as gut signaling, have been shown in animals undergoing TPN therapy [2,3,19,20]. Indeed, a consequence of not having enteral nutrition is intestinal hypomotility which drives bacterial overgrowth and clonal shifts [21].

Several animal studies evaluating morphometric analysis have demonstrated severe gut mucosal atrophy with a significantly lower villous to crypt ratio upon TPN therapy [5,6]. Given this data, it has been postulated and noted in studies that TPN therapy results in increased gut permeability as well as bacterial translocation secondary to such gut injury.

Bacterial permeation, worsened by bacterial overgrowth, drives endotoxin mediated down-regulation of key hepatobiliary receptors and transporters [22]. In clinical studies, new-born babies on TPN have significantly increased susceptibility to TPN associated hepatic injury secondary to endotoxin and bacterial translocation [23].

Several studies have shown an improvement in liver injury after selective antibiotic therapy which points to a direct role of gut bacteria in mediating TPN associated injury [24–26]. Rodent studies have also reported significantly higher Interleukin-6 (IL-6) and Tumor Necrosis Factor (TNF) in animals on TPN in comparison to the control animals [27–29].

Animal studies have shown a dominance of the *Firmicutes* phylum with normal enteral nutrition. Conversely, TPN therapy resulted in a significant proliferation of the pro-inflammatory *Bacteroidetes* phylum, thus altering the normal *Firmicutes* to *Bacteroidetes* ratio. *Bacteroidetes* are known to promote intestinal inflammation [30,31] and increase intestinal permeability [32,33], which can drive bacterial flux across the mucosa and result in cytokine-mediated hepatocellular injury [34–36]. Further highlighting the importance of bile acids and microbiota, enteral bile acid treatment in animals on TPN has been found to preserve clonal gut microbiota shifts and modulate TPN associated injury [20].

Gut microbiota also influence the host immune system. There are several microbial metabolic products such as proteins and polysaccharides that activate recognition receptors such as Toll-like receptors (TLRs) and NOD-like receptors (NODs) which stimulate the mucosal immune system. The gut microbiota in turn promote a tolerogenic state within the intestinal mucosa (stimulating lymphocytes, decreased NF-kB signaling, etc.) and initiates mechanisms to prevent unchecked bacterial overgrowth [7]. Indeed, clonal shifts in the microbiota with TPN therapy are drivers for alteration in immune responses and endotoxin mediated systemic injury.

## Luminal Receptors, Gut Microbiota and TPN

Emerging data also points to modulation of the gut growth regulator TGR5 by gut microbiota in mediating TPN associated injury [19,20,37]. Glucagon-like peptide - 2 (GLP-2), a gut growth hormone, is less activated due to a lack of gut TGR5 activation during TPN [19].

During normal enterohepatic circulation, primary bile acids synthesized hepatically undergo de-conjugation to secondary bile acids by the gut microbiota [38,39].

Preferential activation of FXR is by primary bile acids, while that of TGR5 is by secondary bile acids. Therefore, gut microbiota can shift the bile acid signaling properties through the de-conjugation of primary bile acids [40–42].”

Current evidence also shows that both FXR and TGR5 can modulate the intestinal barrier, permeability, and immune responses [43–46]. One of the key FXR modulated genes is the inducible nitric oxide synthase (iNOS) [45,47], which regulates host immunity as well as antimicrobial activity. Other important FXR modulated angiogenin (ANG1)-coding genes, which are involved in the acute phase response to infection and have potent antibacterial properties, and carbonic anhydrase 12 (CAR12), a host antibacterial defense enzyme [48,49]. Additionally, exogenous activation of TGR5 significantly decreases pro-inflammatory cytokines, specifically interleukin-1α (IL-1 α), IL-1β, IL-6, and TNF-α [50]. Thus, there is significant support for the idea that altered gut microbiota and bile acids during TPN play a prominent role in TPN injury.

## Conclusion

Total Parenteral Nutrition remains a crucial life-supporting therapy. Despite the benefits of TPN, intestinal failure associated liver disease, a disrupted glucose and lipid metabolism, and gut atrophy remain too common. Additionally, enterohepatic circulation and the gut microbiota appear to be altered with TPN, which likely drive the associated liver and intestinal injury. Bile acids in the intestinal lumen, which are ligands for FXR, activate gut FXR to release FGF19 which via portal circulation is delivered to the liver for its metabolic effects. The FGF19-FXR pathway regulates bile acid synthesis, which is disrupted in TPN therapy.

Significant alterations in the gut microbiota have also been reported with TPN use which modulate bile acid deconjugation and in turn the activation of luminal receptors driving hepatic and gut injury. Restoration of microbiota by exogenous bile acids as well as selective enteral antibiotic therapy can mitigate TPN associated injury.

Changes in the intestinal barrier, immune responses and permeability are additional drivers of TPN associated injury.

In summary, there seems to be compelling evidence that gut microbiota and bile acids play an important role in TPN associated injury. Targeting the pathways that regulate such could be key to furthering our understanding of injury associated with TPN and in future therapeutic strategies.

## Abbreviations

PN: Parenteral Nutrition
JV: Jugular Vein
DC: Duodenal Catheters
USDA: United States Department of Agriculture
CDCA: Chenodeoxycholic Acid
BSEP: Bile Salt Export Pump
FGF19: Fibroblast Growth Factor 19
FXR: Farnesoid X Receptor
IFALD: Intestinal Failure Associated Liver Disease
